# Evaluation and physiological response of peony (*Paeonia lactiflora* Pall.) under salt stress

**DOI:** 10.3389/fpls.2026.1739938

**Published:** 2026-05-26

**Authors:** Xiaoyan Lu, Xin Meng, Yichun Hu, Zaisheng Shao, Hengfeng Zhang, Chengzhong Li

**Affiliations:** 1 Department of Landscape Architecture and Horticulture, Jiangsu Agri-animal Husbandry Vocational College, Taizhou, China; 2 Department of Enterprise Management, Jiangsu Sanwei Horticulture Co., Ltd., Suzhou, China; 3 College of Horticulture, Nanjing Agricultural University, Nanjing, China; 4 Ecological Management & Maintenance Center, Nanjing Zhoudao Modern Agriculture Development Co., Ltd., Nanjing, China

**Keywords:** evaluation, morphological indicators, paeonia, physiological response, salt-tolerance

## Abstract

**Introduction:**

Herbaceous peony (*Paeonia lactiflora* Pall.) is widely cultivated in China as a traditional ornamental flower, while soil salinization has negatively affected its cultivation.

**Methods:**

In this study, 20 herbaceous peony cultivars were subjected to 400 mM NaCl solution (1 L per pot), applied once every 3 days for a total of 5 applications over 15 days. A non-saline control (0 mM NaCl) was used for phenotypic reference, with higher phenotypic injury scores representing more serious damage. Seven morphological indices were measured before salt treatment, and phenotypic and physiological indices were determined after treatment.

**Results:**

Phenotypic scoring and cluster analysis indicated that three cultivars, namely ‘Red Charm’, ‘Bartzella’, ‘Sarah Bernhardt’, showed the strongest relative salt tolerance under severe stress. Correlation and stepwise regression analyses suggested that leaf thickness and stem diameter could serve as potential preliminary indicators for salt tolerance evaluation, with higher values corresponding to better salt tolerance. Under salt stress, salt-tolerant cultivars exhibited higher antioxidant enzyme activities, as well as significantly lower relative electrolyte leakage and malondialdehyde (MDA) content than salt-sensitive cultivars (*P* < 0.05). The maximum photochemical efficiency of PSII (*Fv/Fm*) was highly sensitive to salt stress and decreased significantly with reduced salt tolerance (*P* < 0.05).

**Discussion:**

This study provides exploratory evidence for the rapid screening of salt-tolerant germplasm in herbaceous peony using morphological indicators.

## 1 Introduction

Globally, over 900 million hectares of land are affected by soil salinization, which has become a major abiotic factor restricting plant growth and development (Zhou et al., 2024; Shahid et al., 2018; Kumar et al., 2025). Salt stress induces osmotic, ionic, and oxidative stresses that disrupt metabolism, inhibit photosynthesis, and cause growth retardation, chlorosis, or even plant death (Van Zelm et al., 2020; Zhou et al., 2024; Gill and Tuteja, 2010; Al-Janabia et al., 2024). Plant salt tolerance is a complex adaptive trait (Haq et al., 2025; Gong et al., 2020). Plants respond to salt stress through osmotic adjustment, ion compartmentalization, and antioxidant defense systems (Rahman et al., 2021). Although extensive research has been conducted on salt stress in staple crops (Sahito et al., 2024; Ma et al., 2020), forage crops (Feng et al., 2024; Gao et al., 2025), and vegetables (Kumar et al., 2025; Zhao et al., 2017), relatively limited attention has been given to ornamental flowers.

Herbaceous peony (*Paeonia lactiflora* Pall.), belonging to the *Paeoniaceae* family, has a cultivation history spanning over 4,000 years (Li et al., 2020; Wang et al., 2014). It is known for its vibrant colors, large blooms, and strong adaptability, with high ornamental and landscaping value, and is also widely used as a premium cut flower in international markets (Sun et al., 2025; Li et al., 2020). Besides, *Paeonia* possesses remarkable medicinal properties, and its primary bioactive compound, paeoniflorin, exhibits diverse pharmacological effects, including antioxidant, anti-inflammatory, and anticancer activities (Liu et al., 2022; Li et al., 2024). However, salt stress becomes one of the limiting factors for the large-scale cultivation and landscape application of *Paeonia*, as it severely impairs plant growth, development, and ornamental quality. Therefore, a comprehensive understanding of the physiological responses of *Paeonia* under salt stress and evaluation of its germplasm resources is vital for promoting its cultivation.

In recent years, studies have reported the assessment of salt stress in germplasm resources and the screening of various tolerance indicators, mainly using cluster analysis and stepwise regression analysis (Zhao et al., 2024, Zhao et al., 2017). Cluster analysis classifies germplasm based on characteristic similarity, which supports objective grouping. Stepwise regression is appropriate for screening key predictors, as it efficiently selects the most parsimonious set of variables from multiple correlated traits, reducing model complexity. Salt tolerance in sorghum genotypes has been evaluated using indicators such as the K/Na ratio, MDA, MSI, SOD, and proline (Dehnavi et al., 2023). Maximum leaf length, maximum leaf area, relative electrical conductivity, and proline content have been adopted as evaluation indexes for salt tolerance in fennel (Zhao et al., 2024). Chlorophyll fluorescence parameters and SPAD values have also been used to assess salt stress responses in alfalfa seedlings (Zhang et al., 2020). These findings reveal that salt tolerance evaluation indicators vary by plant species, with most studies focusing on physiological indicators. However, measuring these indicators is often complex and time-consuming, making them unsuitable for large-scale, rapid screening. In contrast, morphological indicators offer advantages in operational simplicity and detection speed.

Previous studies on salt tolerance in *Paeonia* have mainly focused on physiological and photosynthetic responses after salt stress. For example, Wang et al. (2013) and Jiang et al. (2018) evaluated physiological changes and salt tolerance levels in several *Paeonia* cultivars, but did not systematically explore morphological indicators for rapid screening. To date, morphological indicators, especially pre-stress morphological traits, have not been rigorously validated for salt tolerance evaluation in *Paeonia* or closely related ornamentals. Therefore, the lack of simple, efficient morphological evaluation indices has become a key bottleneck for rapid and large-scale screening of salt-tolerant *Paeonia* germplasm.

Morphological traits can serve as useful proxies for salt tolerance because they may reflect underlying physiological adaptation mechanisms. In many species, thicker leaves enhance water storage capacity and provide greater volume for ion dilution, thereby mitigating ionic toxicity, while greater stem diameter may indicate improved vascular transport capacity and structural robustness under osmotic stress (Munns and Tester, 2008; Wu et al., 2017; Zhao et al., 2023). For ornamental plants like *Paeonia*, such traits are also directly related to plant vigor and ornamental quality. Thus, these morphological traits are highly promising for early prediction of salt tolerance in *Paeonia*.

To bridge this gap and improve the research system for screening salt-tolerant *Paeonia* germplasm, this study selected 20 *Paeonia* cultivars as experimental materials, and determined their pre-stress morphological indices as well as post-stress physiological indices. The objectives of this study were: (1) to evaluate the relative salt tolerance of these 20 cultivars based on phenotypic changes after a defined severe salt treatment; (2) to screen reliable and easy-to-determine morphological evaluation indices, providing a basis for the rapid identification of salt-tolerant *Paeonia* cultivars; (3) to compare physiological response patterns of *Paeonia* under salt stress and explore the physiological basis associated with its tolerance. We hypothesized that salt tolerance would differ significantly among the 20 *Paeonia* cultivars, and that certain easily measured morphological traits could serve as potential indicators of salt tolerance, and that physiological parameters related to photosynthesis, membrane stability, and antioxidant defense would show consistent response patterns associated with salt tolerance levels.

## 2 Materials and methods

### 2.1 Plant materials

The plant materials are listed in **Table 1**, including 20 *Paeonia* cultivars. All *Paeonia* cultivars were obtained from the National *Paeonia* Germplasm Repository at Yangzhou University. These plants were clonally propagated through division to ensure genetic uniformity. The plants used in this experiment were three-year-old division seedlings that had been potted in April 2023, with one plant per pot. The pots had an upper diameter of 32 cm, a lower diameter of 26 cm, and a height of 35 cm. The cultivation soil was local field soil. The physicochemical properties of the soil were as follows: pH 6.8, electrical conductivity (EC) 184.8 μS cm^-^¹, organic carbon 8.98 g kg^-^¹, total nitrogen 0.98 g kg^-^¹, ammonium nitrogen (NH_4_^+^-N) 0.99 mg kg^-^¹, nitrate nitrogen (NO_3_^-^-N) 15.76 mg kg^-^¹, total phosphorus 0.74 g kg^-^¹, available phosphorus 4.25 mg kg^-^¹, total potassium 16.32 g kg^-^¹, and available potassium 81.5 mg kg^-^¹. The soil texture was sandy loam.

**Table 1:** *Paeonia* cultivars used in this study.

Code	Name	Origin
A	Da Fu Gui	China
B	Mo Yu Lou	China
C	Tao Hua Fei Xue	China
D	Red Charm	U.S.A.
E	He Bao	China
F	Coral Sunset	U.S.A.
G	Zi Feng Yu	China
H	Pillow Talk	New Zealand
I	Mons. Jules Elie	Netherlands
J	Gao Gan Hong	China
K	Hei Hai Bo Tao	China
L	Zi Xiu Qiu	China
M	Bartzella	Japan
N	Hong Mei Gui	China
O	Kansas	Netherlands
P	Xuan Li Duo Cai	China
Q	Qing Wen	China
R	Command Performance	U.S.A.
S	Zhong Sheng Fen	China
T	Sarah Bernhardt	Netherlands

A total of 15 plants per cultivar were initially planted and grown under uniform management. In April 2024 (before salt treatment), nine plants with consistent growth vigor were selected from each cultivar for the formal experiment. Selection was based on visual assessment of plant height, crown width, and branch number, with individuals falling outside the typical range for each cultivar being excluded. Flower buds of all plants were removed to maintain a uniform vegetative growth state during the subsequent salt stress treatment.

### 2.2 Experimental design and treatment

The experiment was conducted in a greenhouse at Jiangsu Agri-animal Husbandry Vocational College (Taizhou, Jiangsu Province, China) under natural light conditions without supplemental lighting. During the experimental period, the photoperiod followed the local natural day-length (approximately 13.5-14 h light/10-10.5 h dark). The average daytime temperature was 25 ± 3 °C, the average nighttime temperature was 18 ± 2 °C, and relative humidity ranged from 60% to 75%. On sunny days, the maximum photosynthetic photon flux density (PPFD) at plant canopy level reached approximately 800-1000 μmol m^-^² s^-^¹.

The experiment was arranged in a completely randomized design (CRD). For each cultivar, three biological replicates were established, with three plants per replicate. All pots were completely randomized at the individual pot level in the greenhouse and rearranged every three days to minimize spatial heterogeneity and edge effects. During the experiment, any emerging flower buds were promptly removed to ensure that all plants remained in a vegetative growth state throughout the treatment period.

Salt stress treatment began on May 9, 2024, designated as Day 0. A non-saline control group (0 mM NaCl) was included. Salt-treated plants were irrigated with 400 mM NaCl solution, while control plants were irrigated with tap water. Starting from Day 0, irrigation was performed every three days between 10:00 AM and 12:00 PM, for a total of five applications until Day 13. Each pot received 1 L of treatment solution or tap water per application, administered slowly in two equal portions to ensure thorough saturation. Any solution that drained into the plastic trays was returned to the corresponding pots to ensure consistent total salt application in the salt-treated group and to avoid uneven water loss in the control group due to differential drainage.

Before salt treatment, morphological indicators were measured for all plants. On Day 15, photographs were taken, and phenotypic traits and physiological indicators were measured for each cultivar. Physiological measurements were performed only on salt-treated plants; the control group served as a visual reference for healthy plant growth under the same experimental conditions.

### 2.3 Measurements

Unless otherwise specified, all morphological and physiological measurements were taken from the 4th to 5th fully expanded, mature functional leaves from the apex. For each plant, one representative leaf at this standardized position was measured, and the value obtained was used as the representative value for that plant. For indicators requiring leaf discs, samples were collected from 2-3 leaves at the same leaf position per plant.

#### 2.3.1 Quantification of phenotypic traits

According to the degree of damage caused by salt stress, and referring to the evaluation system established in previous studies on the physiological and biochemical responses of *Paeonia* under salt stress, the damage was classified into five levels (**Table 2**). Phenotypic scoring was performed by three independent trained evaluators under blinded conditions; evaluators were not informed of cultivar identity during the entire scoring process. Before formal scoring, all evaluators were trained using standardized reference photographs for each damage grade. The final score per plant was calculated as the mean of the three independent scores.

**Table 2:** Evaluation system of the phenotypic traits of the *Paeonia* under salt stress conditions (Wang et al., 2013).

Grade	Description of external morphology	Score
I	No obvious symptoms of damage caused by salt stress	1
II	A small portion of the leaves wilt and droop, or the leaf tips and leaf margins turn yellow	2
III	Approximately half of the leaves show yellowing at the tips and margins, curling and drying	3
IV	Most of the leaves show curling and drying	4
V	The leaves and branches wither until the entire plant dies	5

#### 2.3.2 Morphological indicators

Before salt treatment, plant height, stem diameter, leaf thickness, branch number, leaf length, leaf width, and leaf area were measured. Plant height was measured with a ruler as the distance from the soil surface to the top of the plant. Stem diameter and leaf thickness were measured using a digital vernier caliper with a precision of 0.01 mm; stem diameter was measured at the base of the stem, and leaf thickness was measured on the middle leaflet. Leaf thickness was measured in the central region of the middle leaflet, avoiding the midrib and leaf margin to eliminate the influence of veins and edge effects.

Leaf length, leaf width, and leaf area were measured on a single compound leaf at the 4th to 5th leaf position. Leaf length was measured as the maximum distance from the leaf base (at the petiole junction) to the leaf tip; leaf width was measured as the transverse distance at the widest part of the leaf. Leaf area was calculated using ImageJ software. A scale bar of known length was included in each photograph for calibration. Before measurement, the pixel-to-distance ratio was set in ImageJ using the scale bar, and leaf area was determined using the standard automatic measurement function. Uniform background and lighting conditions were maintained during image capture, and no additional background correction was applied during analysis.

#### 2.3.3 Physiological indicators

SPAD value was measured using a TYS-A chlorophyll meter (Hangzhou Daji Optoelectronic Instrument Co., Ltd., China). For each leaf, three independent readings were taken avoiding the midrib and leaf margin, and the average was used as the representative SPAD value for that plant.

The maximum photochemical quantum yield of photosystem II (Fv/Fm) was measured using a Li-6800 portable photosynthesis system (Li-Cor, USA) between 20:00 and 22:00. During the experimental period, sunset in Taizhou was approximately 19:00, providing at least 60 minutes of natural dark adaptation before measurement; no dark-adaptation clips or additional dark adaptation were applied. Multiple calibrated Li-6800 systems were used with identical standard saturating-pulse settings, and the fluorescence leaf chambers remained darkened throughout the measurement to maintain dark adaptation. Leaf temperature was not actively controlled and was consistent with the ambient greenhouse night temperature (18 ± 2 °C).

Relative electrical conductivity (REC) was calculated as REC (%) = A1/A2 × 100. Fresh leaves were rinsed with deionized water, and leaf discs (1 cm diameter) were collected using a hole puncher. Ten leaf discs were taken per plant, avoiding the midrib and leaf margin. The discs were placed in a syringe with a small amount of deionized water, vacuum infiltrated, transferred to a centrifuge tube containing 20 mL of deionized water, and allowed to stand at room temperature (25 ± 1 °C) for 2 h. The initial conductivity (A1) was measured using a DDS-307 conductivity meter (Leici Instrument Co., Ltd., China). The tube was then boiled for 15 min, cooled to room temperature, and the final conductivity (A2) was measured. REC was measured on a per-plant basis, and the mean value of individual plants within each biological replicate was used for statistical analysis.

Leaf water content (LWC) was calculated as LWC = (WF – WD)/WF × 100%, where WF is fresh weight and WD is dry weight. Leaves were weighed for fresh weight, placed in envelopes, heated at 105 °C for 30 min to deactivate enzymes, then dried at 65 °C to constant weight. After removal from the oven, leaves were cooled to room temperature in a desiccator, and dry weight was measured.

Soluble sugar, soluble protein, malondialdehyde (MDA), superoxide dismutase (SOD) activity, catalase (CAT) activity, and peroxidase (POD) activity were determined using a spectrophotometer and commercial kits (A145-1-1, A045-2, A003-1, A001-1, A007-1-1, and A084-3-1, Jiancheng Bioengineering Institute, Nanjing, China) following the manufacturer’s instructions. Calibration curves were prepared using standards provided in each kit to convert absorbance values to concentrations or activities. Enzyme activities were expressed as units per gram fresh weight (U g^-^¹ FW), and MDA, soluble protein, and soluble sugar contents were expressed as nmol g^-^¹ FW, mg g^-^¹ FW, and mg g^-^¹ FW, respectively. Leaf samples were collected and immediately flash-frozen in liquid nitrogen, then stored at –80 °C until analysis. For extraction, 0.4 g of frozen leaf tissue was homogenized in 1.6 mL ice-cold PBS (pH 7.4) using a pre-chilled mortar and pestle to prepare a 20% homogenate. The homogenate was centrifuged at 4,000 × g for 15 min at 4 °C using a refrigerated centrifuge, and the supernatant was collected for determination. All extraction and measurement procedures were performed on ice or at 4 °C to preserve enzyme activities.

### 2.4 Statistical analysis

The experiment was arranged in a completely randomized design (CRD) with three independent biological replications per cultivar, each replicate containing three plants. The experimental unit was a single potted plant. Data were averaged at the replicate level (n = 3) for all statistical analyses.

Normality of data distribution was tested using the Shapiro-Wilk test, and homogeneity of variances was tested using Levene’s test. One-way analysis of variance (ANOVA) was performed using SPSS 20.0 software (IBM Corp., Armonk, USA). Multiple comparisons of cultivar means were conducted using Duncan’s multiple range test at *P* < 0.05.

Pearson correlation analysis was used to evaluate relationships between morphological indicators and salt-tolerance phenotypic scores. Significance levels are indicated as *P* < 0.05 (*) and *P* < 0.01 (**). Bonferroni correction was applied for multiple comparisons. A complete lower-triangular correlation matrix was generated for all predictors and the phenotypic score.

Hierarchical cluster analysis was performed based on phenotypic scores using squared Euclidean distance and between-group linkage. The optimal number of clusters was determined using the elbow method.

Stepwise regression analysis was conducted using seven morphological indicators as initial predictors. Bootstrap validation with 1000 resamples was performed for the final two-variable model. Regression assumptions (normality, homoscedasticity, linearity, residual independence) were diagnosed. The adjusted R², regression coefficients, and 95% confidence intervals (CIs) are reported. The Durbin-Watson statistic was used.

All figures were generated using WPS Spreadsheets (12.1.0.20305). Data are presented as means ± standard error (SE) of three independent biological replications.

## 3 Results

### 3.1 Morphological indicators of 20 *Paeonia* cultivars before salt treatment

Morphological indicators of plant height, stem diameter, leaf thickness, branch number, leaf length, leaf width, and leaf area of the 20 *Paeonia* cultivars all showed significant differences before salt treatment (one-way ANOVA, *P* < 0.05, **Table 3**). The coefficients of variation for the seven morphological traits ranged from 18.42% to 39.39%, ranked as follows: branch number (39.39%) > leaf area (37.18%) > stem diameter (22.73%) > leaf length (19.80%) > plant height (19.43%) > leaf thickness (18.47%) > leaf width (18.42%).

**Table 3:** Morphological indicators of 20 *Paeonia* cultivars before salt treatment.

Cultivars	Plant height (cm)	Stem diameter (mm)	Leaf thickness (mm)	Branch number	Leaf length (cm)	Leaf width (cm)	Leaf area (cm^2^)
A	43.85 ± 1.90efg†	6.29 ± 0.44fgh	0.23 ± 0.01fg	9.5 ± 0.3bcde	15.70 ± 1.09efg	12.63 ± 0.17ef	69.76 ± 0.46h
B	66.50 ± 3.50a	6.36 ± 0.25fgh	0.24 ± 0.01fg	9.8 ± 0.9abcde	17.90 ± 0.73cde	15.40 ± 0.68abcd	110.57 ± 13.92defg
C	56.85 ± 2.15bc	6.29 ± 0.12fgh	0.28 ± 0.00cde	11.0 ± 1.2abc	17.40 ± 0.67cde	13.58 ± 0.63cdef	108.87 ± 4.74defg
D	37.98 ± 3.85g	8.99 ± 0.78bc	0.32 ± 0.01bc	5.3 ± 1.0fgh	13.73 ± 0.62g	11.70 ± 1.29f	78.58 ± 6.50gh
E	57.55 ± 3.77b	7.70 ± 0.35cdef	0.17 ± 0.01h	9.8 ± 1.0abcde	21.43 ± 0.17ab	14.63 ± 0.64bcde	149.28 ± 15.69bc
F	40.60 ± 1.01fg	10.75 ± 0.71a	0.30 ± 0.01cd	4.3 ± 0.9gh	14.00 ± 0.39g	12.48 ± 1.12ef	67.66 ± 5.11h
G	39.83 ± 4.68g	4.96 ± 0.27h	0.22 ± 0.01g	10.0 ± 1.7abcd	18.50 ± 0.41cde	13.48 ± 0.97cdef	120.67 ± 12.13cdef
H	44.85 ± 2.57defg	6.74 ± 0.33efg	0.26 ± 0.01defg	11.5 ± 0.6abc	14.23 ± 0.46fg	11.48 ± 0.45f	80.47 ± 4.73gh
I	47.68 ± 1.36bcdefg	7.39 ± 0.17def	0.27 ± 0.00def	13.3 ± 0.3a	16.18 ± 0.63efg	12.73 ± 0.88ef	81.19 ± 9.60gh
J	66.65 ± 3.52a	7.87 ± 0.27cde	0.26 ± 0.00defg	8.3 ± 0.9cdef	19.60 ± 1.40bcd	14.58 ± 0.78bcde	135.36 ± 4.81bcd
K	55.03 ± 2.05bc	6.20 ± 0.33fgh	0.26 ± 0.01defg	6.3 ± 1.3efgh	20.30 ± 1.19abc	15.93 ± 0.63abc	153.39 ± 1.68b
L	44.78 ± 2.55defg	6.96 ± 0.37efg	0.26 ± 0.01defg	6.8 ± 1.0defg	18.20 ± 0.45cde	15.13 ± 0.30bcde	137.18 ± 16.19bcd
M	53.35 ± 1.00bcde	7.67 ± 0.30cdef	0.35 ± 0.01ab	7.0 ± 0.8defg	22.60 ± 1.57a	17.88 ± 0.72a	233.92 ± 12.84a
N	50.50 ± 2.26bcdef	5.77 ± 0.22gh	0.24 ± 0.01fg	10.8 ± 1.3abc	17.03 ± 1.00def	13.33 ± 0.43def	107.71 ± 9.59defg
O	54.03 ± 3.53bcd	6.84 ± 0.13efg	0.31 ± 0.02c	6.5 ± 1.2defg	22.00 ± 0.80ab	16.43 ± 1.14ab	128.15 ± 1.46bcde
P	43.18 ± 1.83fg	5.53 ± 0.09gh	0.25 ± 0.01efg	11.5 ± 2.3abc	18.13 ± 1.45cde	12.98 ± 0.98def	99.37 ± 8.60efgh
Q	47.40 ± 2.66cdefg	6.55 ± 0.16efg	0.26 ± 0.01efg	12.0 ± 1.1ab	15.83 ± 1.04efg	12.83 ± 0.06def	94.30 ± 13.94fgh
R	41.80 ± 2.69fg	9.59 ± 1.05ab	0.28 ± 0.00cde	3.0 ± 0.0h	10.78 ± 0.45h	8.73 ± 0.28g	74.70 ± 5.09h
S	54.98 ± 4.77bc	6.22 ± 0.26fgh	0.25 ± 0.02efg	8.8 ± 0.3bcdef	17.33 ± 0.71de	13.20 ± 0.71def	119.08 ± 16.24cdef
T	57.00 ± 4.51bc	8.77 ± 0.88bcd	0.37 ± 0.03a	5.5 ± 0.9fgh	22.70 ± 1.16a	17.7 ± 1.27a	159.47 ± 12.27b
CV(%)	19.43	22.73	18.47	39.39	19.80	18.42	37.18

†The data followed by different letters within a column are significantly different at *P* = 0.05 among different *Paeonia* cultivars.

### 3.2 Changes in phenotypic traits of 20 *Paeonia* cultivars after salt treatment

**Figure 1:**
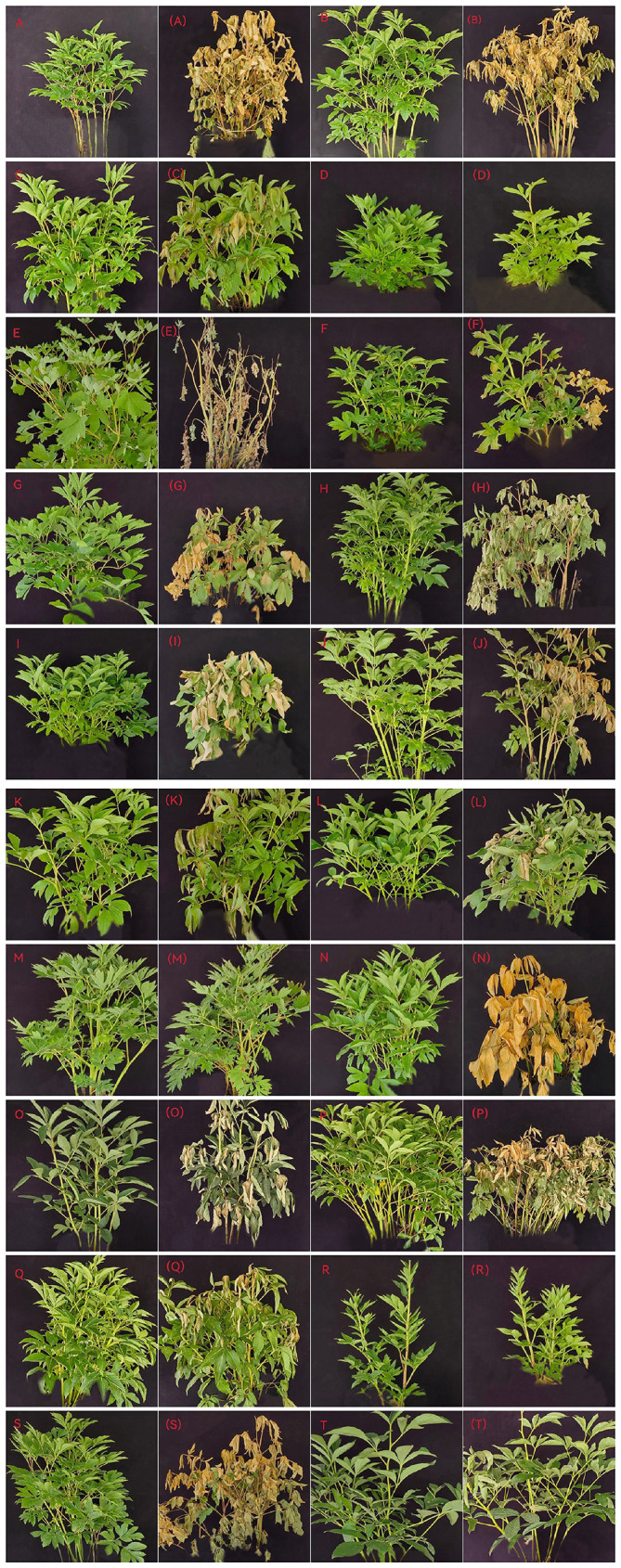
Phenotypic comparison of 20 Paeonia cultivars before and after salt treatment. The letters without parentheses **(A–T)** represent the performance of the corresponding Paeonia cultivar before salt treatment, the letters with parentheses [**(A–T)**] represent the performance of the corresponding Paeonia cultivar after salt treatment.

The phenotypic changes of each *Paeonia* cultivar after salt stress treatment are shown in **Figure 1** and **Table 4**. Cultivars D, M, and T had the lowest scores (1.50 to 1.63), corresponding to Grade I-II damage. In contrast, cultivars A, B, E, N, and S had scores higher than 4.50, corresponding to Grade IV-V damage.

**Table 4:** Phenotypic scores of 20 *Paeonia* cultivars after salt treatment.

Cultivar	Score
A	4.75 ± 0.14a†
B	4.63 ± 0.13ab
C	3.50 ± 0.00cd
D	1.63 ± 0.13h
E	4.88 ± 0.13a
F	3.13 ± 0.13de
G	4.25 ± 0.14b
H	3.50 ± 0.20cd
I	2.88 ± 0.13ef
J	3.50 ± 0.20cd
K	2.25 ± 0.14g
L	2.63 ± 0.13f
M	1.63 ± 0.13h
N	4.50 ± 0.00ab
O	3.13 ± 0.13de
P	3.88 ± 0.13c
Q	3.13 ± 0.13de
R	2.13 ± 0.13g
S	4.50 ± 0.00ab
T	1.50 ± 0.00h

†The data followed by different lowercase letters within a column are significantly different at *P* = 0.05 among different *Paeonia* cultivars.

### 3.3 Cluster analysis of salt-tolerance in 20 *Paeonia* cultivars

**Figure 2:**
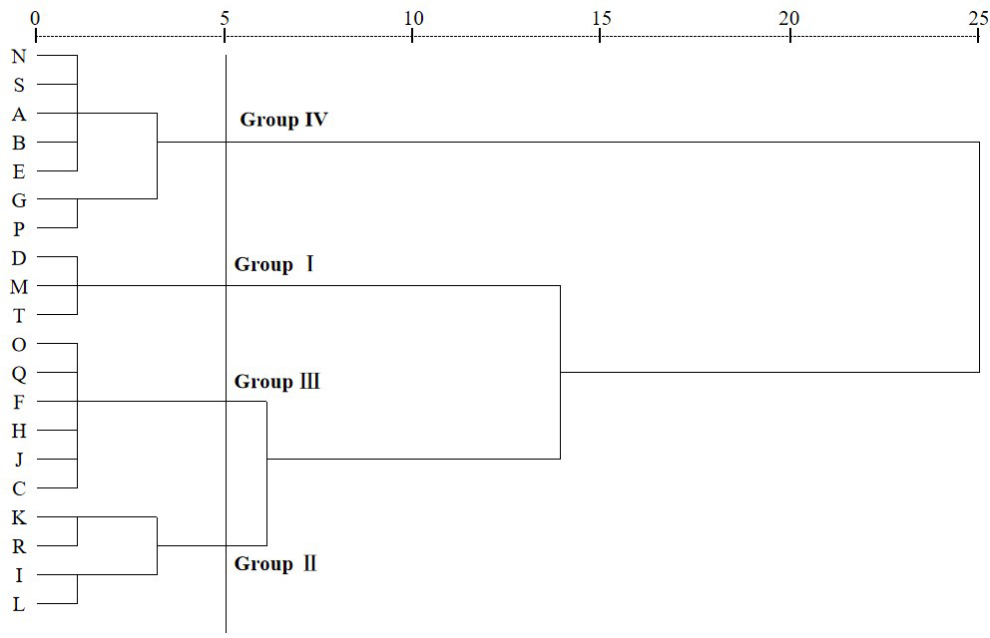
Cluster analysis of the 20 Paeonia cultivars based on phenotypic scores. The numbers of the Y-axis present the code of the Paeonia cultivars; the X-axis presents the Euclidean distance.

Based on the phenotypic scores of the 20 *Paeonia* cultivars, hierarchical cluster analysis was performed using the squared Euclidean distance method and between-group linkage. The elbow method was applied to determine the optimal number of clusters, and the 20 *Paeonia* cultivars were objectively divided into four distinct groups (**Figure 2**):

Group I (cultivars D, M, T): phenotypic scores ranging from 1.50 to 1.63, representing the highest salt tolerance.
Group II (cultivars K, R, I, L): scores ranging from 2.13 to 2.88, representing moderate to relatively high tolerance.
Group III (cultivars O, Q, F, H, J, C): scores ranging from 3.13 to 3.50, representing moderate tolerance.
Group IV (cultivars N, S, A, B, E, G, P): scores ranging from 3.88 to 4.88, representing the lowest salt tolerance.

### 3.4 Correlation analysis between phenotypic scores and morphological indicators of 20 *Paeonia* cultivars

Correlation coefficients between phenotypic scores and seven morphological indicators of 20 *Paeonia* cultivars are presented in **Table 5**. The phenotypic score showed a significantly positive correlation with branch number (r = 0.460, *P* < 0.01), and significantly negative correlations with stem diameter (r = -0.470, *P* < 0.01), leaf thickness (r = -0.734, *P* < 0.01) and leaf area (r = -0.251, *P* < 0.05). No significant correlations were detected between score and plant height, leaf length, or leaf width.

**Table 5:** Correlation analysis between phenotypic scores and morphological indicators of 20 *Paeonia* cultivars.

Indicator	Plant height	Stem diameter	Leaf thickness	Branch number	Leaf length	Leaf width	Leaf area	Score
Plant height	1.000							
Stem diameter	-0.094	1.000						
Leaf thickness	-0.031	0.419**	1.000					
Branch number	0.052	-0.525**	-0.418**	1.000				
Leaf length	0.496**	-0.185	0.054	0.014	1.000			
Leaf width	0.530**	-0.030	0.252*	-0.057	0.819**	1.000		
Leaf area	0.443**	-0.089	0.224*	-0.167	0.781**	0.717**	1.000	
Score	0.151	-0.470**	-0.734**	0.460**	-0.025	-0.170	-0.251*	1.000

*Significant difference at *P* = 0.05.

** Significant difference at *P* = 0.01.

### 3.5 Establishment of regression model and screening of salt-tolerance indicators

To further explore the relationship between seven morphological indicators and salt tolerance in 20 *Paeonia* cultivars, stepwise linear regression analysis was conducted (**Table 6**). With phenotypic score as the dependent variable and morphological indicators as independent variables, the final parsimonious regression equation was:

**Table 6:** Stepwise regression analysis.

Model	Unstandardized coefficients		Standardized coefficients	t	Significance	95% CI for B		VIF
	B	Standard error				Lower	Upper	
(Constant)	8.094	0.488		16.588	0.000	7.122	9.065	
Leaf thickness	-14.452	1.822	-0.652	-7.933	0.000	-18.080	-10.824	1.213
Stem diameter	-0.132	0.055	-0.196	-2.386	0.019	-0.241	-0.022	1.213
*R^2^*	0.571							
Adjusted *R^2^*	0.560							
F	51.250 (*P* < 0.001)							
Durbin-Watson	1.036							

Phenotypic Score = 8.094 - 14.452*Leaf Thickness - 0.132 * Stem Diameter.

Leaf thickness and stem diameter had significant effects on phenotypic score (*P* < 0.05). The model had a coefficient of determination (*R²*) of 0.571 and an adjusted ^R²^ of 0.560, with 95% confidence intervals for the coefficients presented in **Table 6**. Regression diagnostics were performed. The Shapiro-Wilk test yielded *P* = 0.726, the Breusch–Pagan test was not significant (*P* > 0.05), and the Durbin-Watson statistic was 1.036. Bootstrap validation with 1000 resamples for the final two-variable model confirmed the stability of the coefficient estimates.

### 3.6 Physiological response of four different salt-tolerance *Paeonia* groups to salt treatment.

In order to clearly reveal the physiological responses of peonies to salt treatment, the 20 *Paeonia* cultivars were categorized into four groups for analysis based on the results of cluster analysis (**Figure 2**).

**Figure 3:**
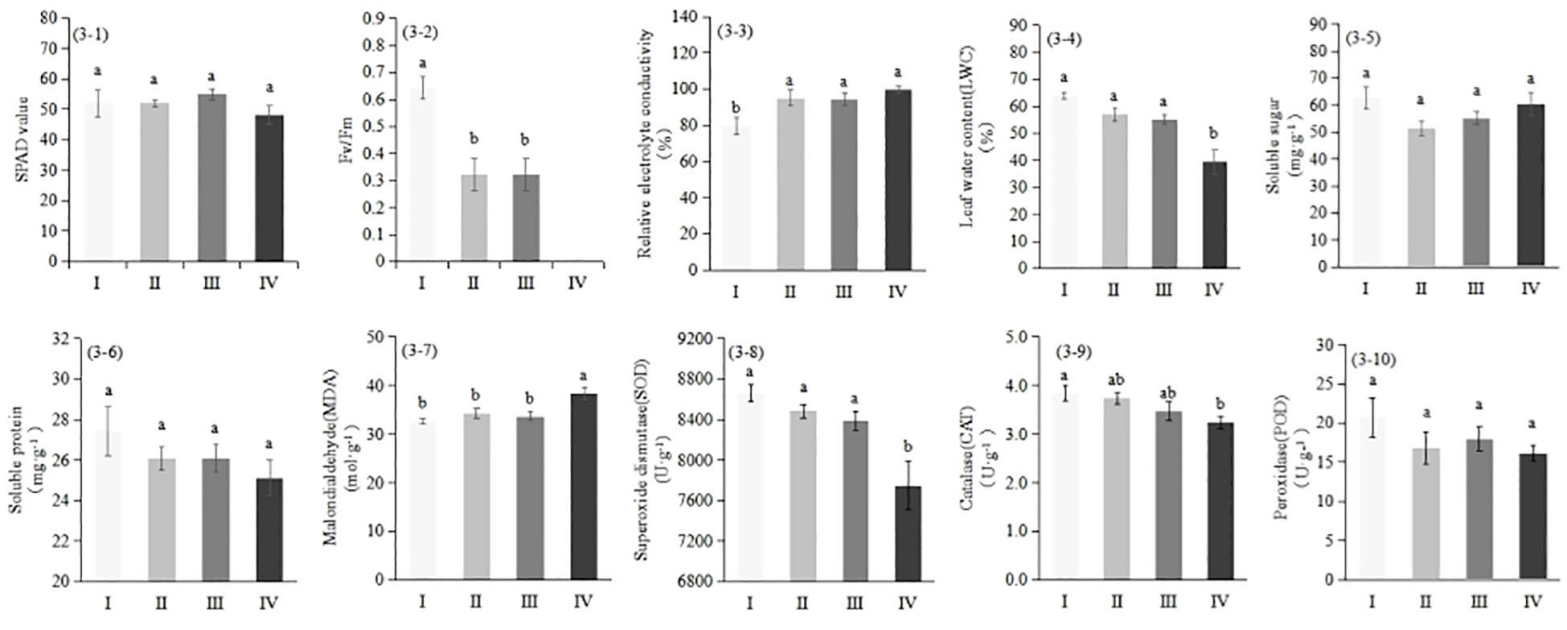
Physiological characteristics of different salt-tolerant groups of Paeonia in the clustering results. (I), Group I; (II), Group II; (III), Group III; (IV), Group IV; (3-1), relative chlorophyll content (SPAD value); (3-2), maximum quantum yield of photosystem II (Fv/Fm); (3-3): relative electrolyte conductivity; (3-4): leaf water content (LWC); (3-5): soluble sugar content; (3-6), soluble protein content; (3-7), malondialdehyde (MDA); (3-8), superoxide dismutase (SOD) activity; (3-9), Catalase (CAT) activity; (3-10), peroxidase (POD) activity. Different lowercase letters within the same index indicate significant differences at P = 0.05. For Fv/Fm (3-2), data for Group IV are not shown because measurements were invalid due to severe tissue necrosis.

The differences in SPAD values among different groups were not significant (**Figure 3-1**). Fv/Fm values were compared among Groups I, II, and III, as measurements for Group IV were invalid due to severe tissue necrosis (no intact green leaf tissue available for reliable measurement). Group I showed significantly higher Fv/Fm values than Groups II and III (*P* < 0.05, **Figure 3-2**). Relative conductivity was significantly lower in Group I than in the other groups (*P* < 0.05, **Figure 3-3**). LWC was significantly lower in Group IV than in the other groups (*P* < 0.05, **Figure 3-4**). The differences in soluble sugar content among different groups were not significant (**Figure 3-5**). No significant differences in soluble protein content were observed among the four groups (**Figure 3-6**). MDA content was significantly higher in Group IV than in the other groups (*P* < 0.05, **Figure 3-7**). SOD activity was significantly lower in Group IV than in Groups I, II, and III (*P* < 0.05, **Figure 3-8**), while CAT activity was significantly lower in Group IV only when compared with Group I (*P* < 0.05, **Figure 3-9**). POD activity showed no significant differences among the groups (**Figure 3-10**).

## 4 Discussion

With the continuous expansion of global soil salinization area, herbaceous peony often suffers from salt stress, which severely inhibits its growth and quality. The evaluation of germplasm resources, particularly the screening of resistant cultivars, is a crucial foundational step in breeding. Based on phenotypic scoring after salt treatment, this study identified three salt-tolerant cultivars, namely ‘Red Charm’, ‘Bartzella’, and ‘Sarah Bernhardt’. The study found that salt-tolerant cultivars exhibited significantly less leaf yellowing and wilting after salt treatment compared with salt-sensitive ones (**Figure 1**). Jiang et al. (2018) also identified four domestic cultivars (‘Fen Yu Nu’, ‘Qi Hua Lu Shuang’, ‘Yin Bian Hong Ge’, and ‘Hong Pan Cai Qiu’) with strong salt tolerance based on phenotypic scores after salt treatment. These cultivars were not included in the present study, and future research should evaluate the salt tolerance of a wider range of cultivars to complete the assessment. This study also revealed that the three selected salt-tolerant cultivars (Group I) had significantly thicker leaves than the salt-sensitive cultivars (Group IV), with stem diameter showing the same trend (**Table 1**). This morphological divergence may be associated with salt tolerance in *Paeonia*. Subsequent stepwise regression analysis supported this relationship, indicating that leaf thickness and stem diameter show potential associations with salt tolerance and may serve as exploratory screening indicators (**Table 6**). Most existing studies rely on physiological indicators measured after salt stress to assess salt tolerance (Kumar et al., 2025; Feng et al., 2024; Jiang et al., 2018). However, the results of this study demonstrate that pre-stress morphological traits also hold significant reference value. This approach offers practical advantages of simple operation, rapid detection, and non-destructiveness, providing a feasible supplementary method for preliminary screening of salt-tolerant *Paeonia* germplasm resources.

In addition, this study also found that there were significant differences in physiological indicators among different *Paeonia* cultivars after salt stress. This is mainly because the physiological and biochemical responses of plants to stress are a dynamic process, and plants can also achieve self-repair through various physiological and biochemical reactions (Hu et al., 2013).

As one of the most stress-sensitive organelles in plants, chloroplasts suffer certain damage under salt stress, which in turn impairs plant photosynthesis (Li et al., 2023). Chlorophyll content is one of the indicators for evaluating plant stress levels. Studies have shown that salt-tolerant cultivars have higher chlorophyll content (Zhang et al., 2017). However, in this experiment, there was no significant difference in SPAD values among *Paeonia* cultivars with different salt tolerance levels (**Figure 3-1**). The reasons for this result may be attributed to the following: SPAD values represent relative rather than absolute chlorophyll content, and in this experiment, the leaves of salt-tolerant varieties were significantly thicker than those of salt-sensitive ones (**Table 3**). This structural characteristic suggests that even if salt-tolerant varieties have higher chlorophyll content per unit weight, the chlorophyll density per unit area (the basis for SPAD measurement) may not show a significant difference. Fv/Fm reflects the maximum potential photochemical efficiency of PSII and is widely used to indicate the degree of damage to the photosynthetic apparatus (Fariduddin et al., 2013). Studies have shown that the Fv/Fm ratio of normally grown plant leaves remains relatively constant after full dark adaptation, generally ranging between 0.80 and 0.85 (Baker, 2008; Zhang and Wang, 2025). Salt stress typically disrupts PSII structure and function, leading to a significant decrease in Fv/Fm. In this study, only Groups I, II, and III were compared because valid Fv/Fm data could not be obtained from Group IV due to severe leaf necrosis. The Fv/Fm values decreased significantly with declining salt tolerance, indicating that salt-sensitive cultivars suffered more serious damage to the PSII system (**Figure 3-2**).

Leaf water content (LWC) is an important physiological indicator for evaluating plant water status and stress response (Kumar et al., 2025; Zhang et al., 2022). The results of this experiment showed that salt-tolerant cultivars maintained significantly higher LWC under salt stress, indicating a stronger water retention capacity (**Figure 3-4**). This physiological characteristic aligned well with the observed phenotypic changes: cultivars with poorer salt tolerance exhibited more severe wilting, corresponding to lower LWC. Malondialdehyde (MDA) content and relative electrical conductivity (REC) are two key indicators for evaluating cell membrane oxidative damage and stress severity (Hamurcu et al., 2020; Weng et al., 2021). In this study, salt-sensitive cultivars (Group IV) exhibited significantly higher MDA content and REC under salt stress (**Figures 3-8**, **Figure 3-3**). Membrane lipid peroxidation not only directly leads to the accumulation of MDA but also impairs the integrity of the cell membrane structure, causing intracellular electrolyte leakage and thereby resulting in increased REC. These findings confirm that salt stress induces severe oxidative damage to the cell membrane of sensitive cultivars, which is consistent with their poor performance under salt stress.

In this study, the activities of the antioxidant enzymes SOD, CAT and POD, which are associated with reactive oxygen species (ROS) scavenging, were generally higher in salt-tolerant cultivars compared to those with poor salt tolerance, with the increases being statistically significant for SOD and CAT (**Figures 3-8**, **Figure 3-9**, **Figure 3-10**). From Group I to Group IV, SOD activity decreased by approximately 10.6%, while CAT activity declined by about 15.7%. These quantitative changes are biologically meaningful, as they are associated with the more severe oxidative damage observed in sensitive cultivars (higher MDA and REC). This finding is in line with the overall conclusion reported by Feng et al. These results suggest that salt-tolerant *Paeonia* cultivars can maintain higher antioxidant enzyme activities under salt stress, which is associated with alleviating oxidative damage and maintaining membrane stability.

Under abiotic stress, plants accumulate osmoregulatory substances, including soluble sugar and soluble protein, to regulate intracellular osmotic balance and protect themselves (Zhong et al., 2019). In this experiment, there was no significant difference in the contents of soluble sugars and soluble proteins among cultivars with different salt tolerance levels. Notably, salt-tolerant *Paeonia* cultivars maintained significantly higher LWC and exhibited stronger antioxidant enzyme activities (e.g., SOD, CAT) under salt stress. These findings suggest that salt-tolerant cultivars may prioritize a robust antioxidant system to protect membrane structure and reduce water loss, thereby effectively maintaining cellular water status. Their salt tolerance may rely more on a synergistic mechanism that integrates water retention and ROS scavenging.

Although this study provides valuable insights into the salt tolerance evaluation of 20 *Paeonia* cultivars through phenotypic and physiological analyses, several limitations should be acknowledged. First, the sample size (20 cultivars) is relatively limited; therefore, the finding that morphological indicators can serve as potential indices for salt tolerance evaluation requires validation in larger, independent germplasm populations to confirm its reliability and generalizability. Second, the use of a single high salt concentration (400 mM NaCl) simulates acute severe stress rather than the chronic salt stress commonly encountered in agricultural production, which limits the ecological relevance and agronomic applicability of the results. Third, the single time-point assessment (day 15) captures only the terminal physiological response at the late stage of stress, failing to reflect the dynamic progression of physiological changes and the pattern of stress acclimation during salt treatment. Fourth, key ion contents (Na^+^, K^+^) were not measured in this study, preventing further inference of core mechanisms underlying salt tolerance formation, such as ion exclusion, ion compartmentalization, and ion homeostasis regulation. Fifth, the stepwise regression model established in this study has not been externally validated, so its predictive ability remains at an exploratory stage without independent verification. To address these limitations, future studies should expand the sample size, establish multi-level salt gradients to simulate field conditions, conduct time-series experiments to capture dynamic physiological responses, measure ion contents to elucidate ion regulatory mechanisms, and perform external validation. These efforts will improve the salt tolerance evaluation system for *Paeonia* and provide a more solid theoretical and practical foundation for the screening and application of salt-tolerant germplasm.

## 5 Conclusion

In this study, 20 *Paeonia* cultivars were classified into four salt-tolerance groups under 400 mM NaCl acute stress using cluster analysis based on phenotypic scores. Three cultivars, ‘Red Charm’, ‘Bartzella’, and ‘Sarah Bernhardt’, were identified as salt-tolerant cultivars, and characterized by relatively thicker leaves and stems. Compared with salt-sensitive cultivars, salt-tolerant cultivars exhibited significantly lower relative electrolyte leakage and MDA content, but a significantly higher Fv/Fm ratio under salt stress. Meanwhile, they maintained higher leaf water content and antioxidant enzyme activities. In addition, an optimal linear regression equation was established using stepwise regression, and leaf thickness and stem diameter were identified from seven morphological indicators as potential indices for evaluating salt tolerance in *Paeonia*. This study provides a preliminary reference for the large-scale screening of salt-tolerant *Paeonia* germplasm resources, although further validation is needed in larger and independent datasets.

## Data Availability

The raw data supporting the conclusions of this article will be made available by the authors, without undue reservation.
